# Mental Health Consultations in Immigration Detention: What Can We Learn From Clinical Records?

**DOI:** 10.3389/ijph.2024.1605896

**Published:** 2024-01-25

**Authors:** Leonel C. Gonçalves, Patrick Heller, Anne-Claire B. Bachmann, Jonathan Barbolini, Clara Fuhrer, Laurent Gétaz, Eric Luke, Hans Wolff, Stéphanie Baggio

**Affiliations:** ^1^ Division of Prison Health, Geneva University Hospitals & University of Geneva, Geneva, Switzerland; ^2^ Institute of Primary Health Care (BIHAM), University of Bern, Bern, Switzerland; ^3^ Division of Prison Health, Geneva University Hospitals, Geneva, Switzerland; ^4^ Faculty of Medicine, University of Geneva, Geneva, Switzerland; ^5^ Division of Tropical and Humanitarian Medicine, Geneva University Hospitals, Geneva, Switzerland; ^6^ Private Practitioner, Geneva, Switzerland

**Keywords:** health care services, immigration detention, mental health consultations, socio-demographic and clinical characteristics, clinical records

## Abstract

**Objectives:** Knowledge on mental health consultations in immigration detention and characteristics of people receiving consultations is scarce. Based on a sample of 230 adult men in immigration detention in Switzerland, we aimed to: (1) Quantify the proportion of persons receiving mental health consultations during detention; and (2) Identify socio-demographic and clinical characteristics associated with mental health consultations.

**Methods:** Retrospective observational study with a cross-sectional design. Prevalence estimates, logistic regressions, and contingency tables were used to analyse the data.

**Results:** A total of 30% of the sample received mental health consultations during detention. Time spent in immigration detention, mental health problems during detention, use of psychotropic medication, and self-harm were associated with mental health consultations. Although mental health consultations are provided to people with more severe mental health problems, 41% of persons with assessed mental health needs during the initial screening and 26% of those who self-harmed during detention did not receive mental health consultations.

**Conclusion:** Mental health resources and screening procedures could be improved to ensure that mental health consultations are matched to clinical need in immigration detention settings.

## Introduction

Conflicts and poverty have increased the number of displaced people [1] and stringent measures are frequently applied to these persons, including detention for migration-related reasons. Indeed, the use of immigration detention has augmented worldwide in the previous 10 years [1, 2]. Worryingly, undocumented migrants—who are a vulnerable group of persons due to pre- and post-migration factors—are often detained for a long time under restrictive conditions [3]. For instance, the European Committee for the Prevention of Torture and Inhuman or Degrading Treatment or Punishment raised concerns about the living conditions under immigration detention in European countries (e.g., limited access to telephone, Internet, outdoor activities, and exercise) and the provision of mental healthcare [4–7].

People in immigration detention carry a severe burden of mental health problems—especially internalizing disorders such as depression, anxiety, and post-traumatic stress disorder (PTSD)—that tend to be aggravated during incarceration [2, 8]. Recent meta-analyses estimated that the overall prevalence of these mental health disorders in immigration detention ranged between 54% and 65% for anxiety, 68% and 74% for depression, and 42% and 46% for PTSD [9, 10]. To date, only one empirical study has focused on mental health in immigration detention in Switzerland. The study found that 76% of the persons detained in the Basel detention center had at least one psychiatric disorder [11]. This prevalence rate is almost twice than among non-detained asylum seekers in the canton of Zurich, where it was found that 40% met criteria for a mental health disorder, with prevalence rates of anxiety, depression and PTSD ranging from 8% to 31% [12].

Although there are some studies on the prevalence of mental health problems in immigration detention, empirical research on the provision of mental health consultations in this context are very scarce. However, testimonies from persons in immigration detention indicate that responses to their emotional distress are often inappropriate or inconsistent, and that quality of care tend to be superior in regular prisons [13–15]. A study conducted in Australia [16] revealed that 50% of persons in immigration detention attended the emergency department of an hospital in a year, the most common diagnoses being related to psychiatric problems (24%). Among (non-detained) asylum seekers in Switzerland, it was found that only 26% had received any kind of psychiatric treatment in a 12-month period [17].

Moreover, to the best of our knowledge, there is no empirical study on the characteristics of persons receiving mental health consultations during immigration detention. It is known that persons who stay longer in immigration detention [18–21] and who have been exposed to traumatic events [22–24] tend to have more mental health problems. However, it is unknown if psychiatric treatment matches mental health needs of persons in immigration detention.

Besides being scarce, prior studies on mental health in immigration detention carry limitations. Most are narrative or descriptive and come from English-speaking countries. In addition, empirical studies generally rely on small sample sizes and include restrictive inclusion criteria. Additionally, results differ considerably across countries and from one detention center to another, related to mental health practices and availability of services in different institutions [25].

Research in immigration detention is also limited because of logistical issues. It includes, for example, participants’ inability to speak the country’s language, the lack of validated instruments in foreign languages, their short duration of detention or the lack of funding to conduct high-quality exploratory research in such settings. Because of these issues, conducting prospective studies in immigration detention may be very challenging. An alternative is to use retrospective studies, relying on clinical records. Even if this strategy has important limitations, such as the lack of standardized tools and identification of more acute cases, it has other strengths. For example, information on medications and healthcare visits are available, improving our understanding of people’s mental health services. Overall, such retrospective studies may help overcoming research barriers.

In sum, despite the growing number of persons incarcerated for migration-related issues, studies on immigration detention are scarce and limited in scope. Attending to the high burden of mental health problems among persons in immigration detention, more knowledge regarding the psychiatric treatment of this population and its adequacy is crucially needed to assure their human rights. Based on the clinical records of persons in immigration detention in Switzerland, we aimed to [1]: quantify the proportion of persons receiving mental health consultations during detention; and [2] identify socio-demographic and clinical characteristics associated with mental health consultations. The results of this study may allow to develop recommendations for action in daily practice, namely the treatment needs of specific groups and the necessary allocation of resources for psychiatric treatment.

## Methods

### Setting

The study took place in the Canton of Geneva, Switzerland. In Switzerland, immigration detention can last up to 18 months [26–29]. In 2018, 3,284 cases of immigration detention were ordered, the average duration of detention was 25 days, and the annual costs amount to 20 million Swiss francs [26]. Immigration detention in Switzerland has been severely criticized (e.g., abusive implementation, absence of the right to legal representation, lack of adapted infrastructures, excessive restriction of movements, insufficient occupational and recreational activities, and insufficient provision of mental health services [26, 28–31].

There are two immigration detention centers in the canton of Geneva, with 20 places each. At entry in immigration detention, a medical screening is conducted by a nurse. General practitioners and forensic psychiatrists provide weekly medical ward rounds and are available for emergencies. All acute psychiatric issues involving behavioral problems (e.g., self-harm events, harm to others, acute uncontrolled psychiatric symptoms, need of psychiatric hospitalizations) are taken care by the clinical team and are therefore registered in clinical records.

### Procedure

This was a retrospective observational study with a cross-sectional design. Data were collected by medical students between July and December 2019, using medical and institutional files. Data were manually extracted from the clinical records. The Geneva’s cantonal ethics committee approved the study protocol (no. 2020-01323).

### Variables

*Mental health consultations* represent the number of consultations provided to address mental health problems during immigration detention and were counted from the electronic database used by the medical services. Mental health consultations are provided based on the necessity established by medical staff or at request of the detained persons. These services can be provided by psychiatrists but also by medical doctors and nurses. The initial screening of the detained persons made by the nurse was not counted as a consultation. The number of mental health consultations was dichotomized for analyses because most persons had no consultation for the treatment of mental health problems.

*Socio-demographic variables* included age, region of origin (Europe, Africa, Asia, and America), and the number of days incarcerated in the current institution.

*Clinical variables* included having a history of alcohol or/and drug addiction, having received mental health treatment before detention, and traumatic health problems (i.e., physical problems related to accidents and violence).

*Mental health variables* included having mental health symptoms during detention and were assessed first by certified psychiatrists (EL, PH). The psychiatrists reviewed a second time each case, supported by psychologists (LCG, SB). Mental health problems did not represent mental health disorders as established by diagnostic tools, but rather clinical evaluations of the psychiatrists based on the case note information and their knowledge of the patients. The cases review allowed standardization of mental health problems in the study sample. Binary variables indicating the presence of any mental health problem and their comorbidity (more than 1 mental health symptom) were created. In addition, we identified persons who were referred as potentially having any type of mental health problems during the initial screening, received psychotropic medication, and committed self-harm during detention (i.e., suicide attempts, auto-mutilations, and hunger strikes).

### Statistical Analyses

We determined the appropriate sample size to estimate the proportion of persons receiving mental health consultations in immigration detention in Switzerland. With a margin of error of 5%, a confidence interval of 90%, a population of 4801 persons (average number of persons in immigration detention in the country [32], and a proportion of 26% (based on the proportion of treated asylum seekers in the community) [17], the required sample size was 200 persons [33].

To quantify the proportion of persons receiving mental health consultations in immigration detention (objective 1), we used estimates of means along with confidence intervals (CI). To identify characteristics associated to mental health consultations (objective 2), the outcome was regressed on socio-demographic, clinical, and mental health variables using hierarchical logistic regressions. Model 1 presented the association between mental health consultations and socio-demographic characteristics, Model 2 added clinical variables, and in Model 3 mental health variables were included. Besides that, we used contingency tables to compare the proportion of detained persons who received mental health consultations and those who did not, among persons having mental health needs. There was no missing data. Statistical analyses were conducted with Stata 17.

## Results

### Sample

The study sample included 230 males (*n* = 120 detained in Frambois and *n* = 110 detained in Favra), with a mean age of 31 years (range 18–56). Their time spent in immigration detention (for the current stay) spanned an average of 29 days (range 1–232). Most persons came from the African continent (63.0%). At the clinical level, 37.4% had a history of alcohol and/or drug abuse and 23.9% had received prior mental health treatment. The complete list of descriptive characteristics can be seen in Table 1.

**TABLE 1:** Descriptive characteristics of the sample (Geneva, Switzerland, 2023).

Variable	Mean/prop.	SD/*n*	Min.	Max.
Age	30.9	8.8	18	56
Europe	25.7	59	0	1
Africa	63.0	145	0	1
Asia	07.4	17	0	1
America	03.9	9	0	1
Days in immigration detention	29.4	40.6	1	232
Alcohol/drugs history	37.4	86	0	1
Prior mental health treatment	23.9	55	0	1
Traumatism/violence	29.1	67	0	1

Note. Prop., proportion; SD, standard deviation; *n*, partial sample size; Min., minimum; Max., maximum.

### Mental Health Problems and Consultations

Table 2 presents the proportion of persons receiving mental health consultations and having mental health problems. The results revealed that 30.0% of the sample have had mental health consultations.

**TABLE 2:** Proportion of mental health problems and consultations (Geneva, Switzerland, 2023).

Variable	Prop.	CI
Mental health consultations	30.0	[24.4, 36.3]
Mental health problems at screening (nurse)	24.3	[19.2, 30.3]
Mental health problems during detention (psychiatrists)	28.3	[22.8, 34.5]
Comorbidity of mental health problems	09.6	[06.4, 14.1]
Psychotropic medication	36.1	[30.1, 42.5]
Self-harm	11.7	[08.2, 16.6]

Note. Prop., proportion; CI, 95% confidence interval.

Based on the screening made by the nurses, 24.3% had some type of mental health issue at entry. The evaluation of the psychiatrists indicated that 28.3% had discernible mental health problems during detention and that 9.6% had more than one type of symptom. Psychotropic medication was prescribed to 36.1% of the sample. In addition, 11.7% committed acts of self-harm during immigration detention.

### Characteristics Associated With Mental Health Consultations

The characteristics associated with mental health consultations in immigration detention are presented in Table 3. Among socio-demographic characteristics (Model 1), a longer time spent in immigration detention was significant (*p* < .001). When including clinical variables (Model 2), having received mental health treatment before detention (*p* < .001) and a history of alcohol/drug dependence (*p* = .045) were associated with mental health consultations. Time in immigration detention remained significant (*p* < .001) and this model better predicted the outcome (Pseudo *R* ^2^ 24.4% vs. 11.7%). The last step (Model 3) added the variables related to mental health during detention. Having mental health symptoms (*p* < .001), receiving psychotropic medication (*p* = .002), and self-harm behaviors (*p* = .036) were associated with mental health consultations. Time in immigration detention (*p* = .004) remained associated with the outcome but a history of alcohol/drug dependence and mental health treatment before detention lost significance. Model 3 was considerably better than Model 2 (Pseudo *R* ^2^ 65.2% vs. 24.4%).

**TABLE 3:** Characteristics associated with mental health consultations (Geneva, Switzerland, 2023).

Variable	Model 1			Model 2			Model 3 (%)		
	*b*	CI	*p*	*b*	CI	*p*	*b*	CI	*p*
Age	0.02	[−0.01, 0.06]	.222	0.03	[−0.01, 0.07]	.191	0.03	[−0.03, 0.09]	.276
Europe (comparison group)	—	—	—	—	—	—	—	—	—
Africa	0.55	[−0.29, 1.39]	.199	0.22	[−0.72, 1.15]	.647	0.35	[−1.22, 1.92]	.663
Asia	−0.02	[−1.44, 1.40]	.974	−0.38	[−1.93, 1.17]	.633	−0.58	[−3.19, 2.02]	.660
America	0.76	[−0.88, 2.39]	.366	1.25	[−0.47, 2.96]	.154	2.48	[−0.15, 5.11]	.064
Days in immigration	0.02	[0.01, 0.03]	<.001	0.02	[0.01, 0.03]	<.001	0.02	[0.01, 0.03]	.004
Alcohol/drugs history	—	—	—	0.75	[0.02, 1.49]	.045	−0.59	[−1.91, 0.72]	.377
Prior mental health treatment	—	—	—	1.65	[0.88, 2.41]	<.001	−0.31	[−1.85, 1.23]	.691
Traumatism/violence	—	—	—	0.28	[−0.47, 1.02]	.467	0.06	[−1.25, 1.36]	.929
Mental health problems at screening	—	—	—	—	—	—	0.95	[−0.22, 2.12]	.111
Mental health problems during detention	—	—	—	—	—	—	3.66	[2.37, 4.95]	<.001
Comorbidity of mental health problems	—	—	—	—	—	—	−0.20	[−2.28, 1.87]	.848
Psychotropic medication	—	—	—	—	—	—	2.53	[0.95, 4.10]	.002
Self-harm	—	—	—	—	—	—	1.87	[0.12, 3.62]	.036
Pseudo *R* ^2^	11.7%			24.4%			65.2		

Note. *b*, unstandardized regression coefficient; CI, 95% confidence interval; *p*, statistical significance.

The contingency table associating mental health variables and consultations are presented in Table 4. Although there were significant differences between groups (all *p* < .001), still, 41.1% of the persons referred as having mental health needs at screening, 13.9% identified as having mental health problems by the psychiatrists, 9.1% with comorbidity of symptoms, 28.9% receiving psychotropic medication, and 25.9% who committed acts of self-harm did not receive any mental health consultation during detention.

**TABLE 4:** Association between mental health variables and consultations (Geneva, Switzerland, 2023).

Variable	Mental health consultations *n* (prop.)	
	No	Yes
Mental health problems at screening	23 (41.1)	33 (58.9)
Mental health problems during detention	9 (13.9)	56 (86.2)
Comorbidity of mental health problems	2 (09.1)	20 (90.9)
Psychotropic medication	24 (28.9)	59 (71.1)
Self-harm	7 (25.9)	20 (74.1)

Note. *Prop., proportion; n*, partial sample size.

## Discussion

To develop knowledge on mental healthcare in immigration detention, this study aimed to quantify the proportion of persons receiving mental health consultations during detention and identify characteristics associated with these services. The results showed that most persons had no mental health consultation during their time in immigration detention in Switzerland. Time spent in the institution and acute mental health problems were the variables most related with mental health consultations. However, 41% of the persons referred as having mental health needs at screening, and 26% of those who self-harmed did not receive any mental health consultation during immigration detention.

Having a closer look on the association between mental health variables and the provision of mental health consultations (Table 4), we found that most persons having mental health needs received treatment. However, especially among persons identified as potentially having mental health problems at screening and those who committed acts of self-harm during detention, a large proportion (41% and 26%, respectively) had no mental health consultation. Besides, mental health screening was not associated with psychiatric services, when taking other variables into account (see Table 3). This is surprising because persons identified as having mental health needs at entry in immigration detention are generally redirected to mental health professional, independently of their actual mental health status [34]. In practice, this is explained by the lack of psychiatric resources and the fact that primary care physicians take care of these patients with nurses. Nevertheless, perhaps the screening procedure could be improved to ensure that mental health consultations are matched to clinical need [35, 36].

The proportion of persons receiving mental health consultations was 30% (Table 2). This is slightly higher than found in immigration detention in Australia (24%) [16] and among non-detained asylum seekers in Switzerland (26%) [17], although the contexts and time frames are not directly comparable. Nevertheless, this proportion may be lower than among the general prison population of the country. A study among 1,664 adults detained in the Canton of Vaud estimated that 43% received mental health consultations [37]. The same rate (43%) was found in a study among 7,965 persons detained in Canadian federal prisons [34]. The lower rate observed in our study can be explained by the shorter time persons stay in immigration detention when compared to regular prisons. However, it can also indicate reduced mental health resources in immigration detention. Considering that about 76% of people in immigration detention in Switzerland have a psychiatric disorder [11], a consultation rate of 30% is quite low.

Regression analyses (Table 3) revealed that a longer time spent in immigration detention, having mental health problems during detention, receiving psychotropic medication, and committing acts of self-harm were the variables more associated with mental health consultations. Among these covariates, mental health problems had the strongest effect on the outcome, which suggests that treatment is indeed provided to persons who have mental health needs. However, it must be noted that, with the methodology of this study (based on clinical records), only 28% of the sample were found to have mental health symptoms. This corresponds to the most acute cases detected by mental health professionals with the available information. Differently, studies made on the basis of standardized diagnostic tools evidence higher prevalence rates because they provide a more throughout assessment of the persons. Therefore, the rate found in this study is probably an underestimation. What can be concluded based on the current findings is that mental health consultations in immigration detention is provided to the persons who have more serious mental health problems.

It is expectable that persons who received psychotropic medication tend to have mental health consultations, since the prescription of drugs generally requires a psychiatric assessment. Persons receiving psychotropics without having mental health consultations are mostly those to whom opiates are prescribed because when they are transferred from other institutions many already have a treatment program for drug addiction and continue receiving their medication without having an immediate assessment. In addition, most persons who committed acts of self-harm during detention received mental health consultations because they generally represent emergency cases requiring an immediate intervention from the medical services of the institution. In this regard, it is worrying that almost 12% of our sample self-harmed during their (short) stay in the institution, which highlights the high proportion of persons with acute distress in immigration detention. Similarly, other studies evidenced high rates of self-harm among persons in immigration detention, ranging from 13% to 22% in England and Australia [38, 39].

In addition, persons who spent more time in immigration detention were more likely to receive mental health consultations. This can happen because mental healthcare staff have more opportunity to identify and treat persons who stay longer in the institution (in our sample, almost 37% were detained for less than 1 week), and the cross-sectional design of our study does not allow to control for the length of exposure to the outcome. However, several studies have evidenced the negative effect of time spent in immigration detention on mental health [18–22, 38, 40] and it is known that the prevalence and severity of symptoms are higher in detained relative to non-detained samples of undocumented migrants [2, 8, 10, 18, 24]. Therefore, it is possible that persons in immigration detention develop more mental health problems requiring treatment the longer they stay in the institution.

### Limitations and Implications

The results of this study should be interpreted at light of its limitations. First, although reflecting the practices of the research sites, our assessment of mental health symptoms is an underestimation the true rate of mental health problems. Furthermore, it is difficult to compare our estimates with those of other studies due to different research methodologies, cultural and population peculiarities, and the practices of each institution [25]. Therefore, the prevalence rates presented here cannot be considered representative of immigration detention in Switzerland or elsewhere. In addition, the set of variables identified in this study is not exhaustive and we might have missed important covariates of mental health consultations (e.g., having experienced past traumatic events, abuse and neglect). Finally, due to the retrospective cross-sectional design of the study, no causal associations between variables can be made.

Despite limitations, this study has implications for research and practice. Although not representative of Switzerland, the mental health consultation rate found in this study (30%) can be used to compare the treatment rate in other immigration detention settings. Furthermore, mental health services appear to focus on persons with more severe mental health problems, but many persons with mental health needs do not receive mental health consultations and not all services are provided by psychiatrists or psychologists. Increasing the number of specialized mental health staff would allow to provide more adequate services to persons in immigration detention. In addition, the mental health of persons in immigration detention may deteriorate over time in detention and require more mental healthcare. Diverting undocumented migrants to community institutions and using detention for the shorter amount of time could help to prevent the detrimental effect of incarceration on mental health and save medical resources. Considering the high rate of self-harm in immigration detention, this measure should be used as a last resort. Finally, including standardized diagnostic tools for the triage of persons with mental health problems in the initial screening made by the nurses could help for the early identification and treatment of persons with psychological difficulties.

To conclude, the burden of mental health problems among undocumented migrants is high and immigration detention can further affect their mental health. Although focusing resources on acute cases, many persons with mental health needs in immigration detention may not be identified or receive mental health consultations, and treatment policies may not be the most adequate. More studies on mental health needs and services in immigration detention settings are needed to understand how to improve the cost-effectiveness of psychiatric services for persons with migration-related issues. This is necessary to guarantee their human rights and prevent the negative impact of custody.
